# Treatment with rapamycin prevents induction and expression of locomotor sensitization to synthetic cathinone 3,4-methylenedioxypyrovalerone (MDPV) in mice

**DOI:** 10.1007/s11419-025-00749-w

**Published:** 2025-11-27

**Authors:** Jakub Wojcieszak, Katarzyna Kuczyńska, Jolanta B. Zawilska

**Affiliations:** Department of Pharmacodynamics, Medical University of Lodz, Muszyńskiego 1, 90-151 Łódź, Poland

**Keywords:** MDPV, Addiction, Sensitization, Rapamycin, Synthetic cathinones

## Abstract

**Purpose:**

3,4-Methylenedioxypyrovalerone (MDPV) is a potent psychostimulant substance endowed with addictive properties. As mammalian target of rapamycin (mTOR) mediates neuroadaptive changes responsible for development of addiction, the current study evaluated whether rapamycin, a potent and selective inhibitor of mTOR, prevents induction and expression of behavioral sensitization in mice treated with MDPV.

**Methods:**

Locomotor sensitization was used as an animal model of early phase of addiction. C57BL/6JRj mice were treated with rapamycin before administration of MDPV during the induction phase of sensitization, or during the final 5 days of the withdrawal. Sensitization was assessed based on the measurement of locomotor activity after treatment with MDPV.

**Results:**

Rapamycin administered on days 1–7 inhibited induction of sensitization characterized by increased horizontal and vertical locomotor activity on day 7 compared to day 1. Additionally, when given during the withdrawal from MDPV, rapamycin blocked expression of sensitization, defined as augmented response to MDPV on day 21 compared to day 1.

**Conclusions:**

Abolishment of locomotor sensitization to MDPV by rapamycin suggests that neuroadaptive changes underlying this phenomenon are dependent on the mTOR signaling and warrants further research on possible application of mTOR inhibitors in treatment of addiction.

## Introduction

Synthetic cathinones (SC) are one of the most prevalent groups of new psychoactive substances (NPS), which emerged on the illicit drug market and gained popularity in the second half of 2000s [1, 2]. These compounds were synthesized by modification of the scaffold of a naturally occurring alkaloid, cathinone [2]. Due to structural similarity to monoamine neurotransmitters, SC can interact with respective transporter proteins: dopamine transporter (DAT), norepinephrine transporter (NET) or serotonin transporter (SERT), leading to inhibition of monoamines uptake or to increase of their release into the synaptic cleft. Via this molecular mechanism of action, SC produce psychostimulant effects similar to those of old drugs of abuse, such as amphetamine, methamphetamine or cocaine [2–4]. Like classic psychostimulants, SC may induce physical addiction upon repeated intake [2]. It has been documented that addictive potential of SC, as well as other psychostimulants, is positively correlated with their potency to stimulate dopaminergic neurotransmission in brain structures, such as striatum, and with a selectivity for DAT over SERT [2–5].

3,4-Methylenedioxypyrovalerone (MDPV) belongs to α-pyrrolidinophenone derivatives, a subgroup of SC, which is characterized by potent inhibition of DAT and NET, resulting in very pronounced psychostimulant effects [3, 5, 6]. MDPV is 10- to 50-fold more potent than cocaine as a DAT blocker [6], and exerts powerful rewarding and reinforcing effects relative to cocaine at one-tenth the dose. Because of its pharmacological profile, MDPV bears highly addictive properties, which were documented in human abusers [7] as well as in behavioral rodent paradigms, such as self-administration [8, 9], drug discrimination [10], behavioral sensitization [11–14] or conditioned place preference [12, 15]. In recent years abuse of SC and other NPS have become increasingly popular [1]. This raises concerns among healthcare specialists about associated risks, including acute toxicity, overdose and addiction. Importantly, accumulating evidence indicate high health risks related to use of MDPV, as demonstrated by cases of non-fatal and fatal intoxications [7, 16–19]. Therefore, increased efforts to counteract these threats are necessary. At present, the knowledge on direct molecular mechanism(s) of the development and expression of addiction to psychostimulants is limited. Likewise, the availability of efficient treatment options for addicted individuals is not satisfactory. As SC appeared on the illicit drug market relatively recently, fewer studies have been conducted and published about their addictive properties in comparison to old drugs of abuse. Most studies performed on laboratory rodents exploring addictive potential of cathinones focused on their direct interactions with monoamine transporters and induction of changes in monoamine signaling, such as changes in expression of DAT or D1:D2 dopamine receptors density ratio, and used anti-dopaminergic treatments to reverse rewarding effects of SC [9, 12, 20–23].

The mammalian target of rapamycin (mTOR), also referred to as the mechanistic target of rapamycin, is a 289-kDa serine-threonine kinase that belongs to the phospho-inositide 3-kinase (PI3K)-related kinase family. mTOR forms two multiprotein complexes, mTORC1, which is sensitive to rapamycin, and mTORC2, which is not directly inhibited by this drug. mTOR complexes are composed of discrete protein binding partners to regulate numerous cell functions. The main function of mTORC1 is to regulate protein synthesis and cell growth through downstream molecules, i.e., eukaryotic initiation factor 4E-binding protein1 (4E-BP1) and p70 ribosomal S6 protein kinase (S6K), while mTORC2 plays an important role in a growth factor signaling by phosphorylating the C-terminal hydrophobic motif of some AGC kinases, like Akt and SGK [24]. The mTORC1-controlled signaling pathways regulate many functions of the nervous system, including neuronal development, synaptic plasticity, memory storage, and cognition. Of note, it has been previously found that cocaine induces mTORC1 activity in addiction-related brain structures, such as nucleus accumbens (NAc), ventral tegmental area (VTA) and prefrontal cortex (PFC), where it is responsible for adaptive changes leading to sensitization and drug seeking [25–30]. In line with that rapamycin, a selective inhibitor of mTORC1, was found to suppress behavioral sensitization to cocaine and methamphetamine without affecting acute locomotor response to these drugs [25, 26, 30, 31] as well as other behavioral markers of addiction in rodents, including conditioned place preference and reinstatement of drug-seeking in self-administration paradigm [32–34].

In the current study, we aimed to verify whether rapamycin could successfully block sensitization to MDPV, a more potent and addictive drug than cocaine. Behavioral sensitization paradigm was used as a behavioral model for an early stage of addiction to the drug [11]. To the best of our knowledge, this is the first study aiming to elucidate the effect of inhibition of mTOR by rapamycin on the behavioral sensitization to SC, using MDPV as a model representative of the group.

## Materials and methods

### Animals and treatments

All housing conditions and experimental procedures were in accordance with the European Union guidelines regarding the care and use of laboratory animals (European Communities Council Directive of September 2010 (2010/63/EU)) and ARRIVE guidelines [35]. All efforts were made to minimize animal suffering and to reduce the number of animals used.

All procedures were conducted using male C57BL/6JRj mice (Janvier Labs, Le Genest-Saint-Isle, France). Mice were 9 weeks old upon arrival to the animal facility and were housed in groups of four per standard cage (cage height 14 cm, minimal surface/mouse = 132.5 cm^2^), enriched with one tunnel, wooden block for biting and nesting material, with ad libitum access to tap water and standard chow. Cages were kept in a sound-attenuated room with automatic 12-h light/dark cycles (lights on at 6:00 a.m.), at temperature of 20–24 °C and relative humidity of 45–65%. Experiments started after 1 week of a mandatory quarantine.

**Fig. 1:**
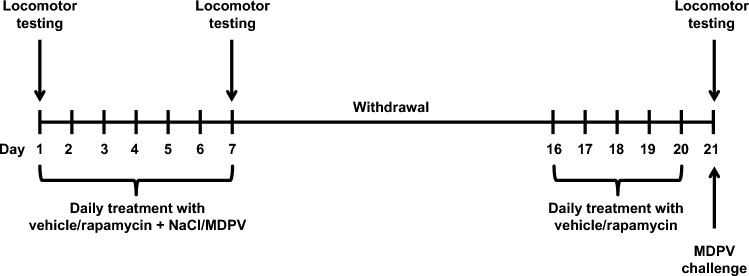
Schematic representation of the experimental design

Experimental timeline is presented on Fig. 1. Mice were randomly divided into four groups, each consisting of 12 animals. Treatment schedule is presented in Table 1. During days 1–7 each mouse received two i.p. injections. Firstly, the animals received either rapamycin (10 mg/kg bw, 0.1 mL/10 g bw; MedChemExpress, Monmouth Junction, NJ, USA) in a vehicle consisting of 2% ethanol, 5% Tween-80, 5% PEG400 in sterile water for injections (group 3) or vehicle (groups 1, 2, 4). After 1 h, mice received s.c. injection (0.1 mL/10 g bw) of either 0.9% sterile saline (group 1) or MDPV (1 mg/kg bw; LGC, Luckenwalde, Germany) dissolved in sterile saline (groups 2, 3, 4). Then the animals underwent a 13-day withdrawal from MDPV (days 8–20). During the last five days of withdrawal (days 16–20) mice were injected i.p. (0.1 mL/10 g bw) with either vehicle (groups 1, 2, 3) or rapamycin (10 mg/kg bw; group 4). On day 21 all mice were challenged with MDPV (1 mg/kg bw, s.c.).

**Table 1:** Schematic representation of the treatment schedule

	Group 1	Group 2	Group 3	Group 4
Day 1–7	Vehicle + Saline	Vehicle + MDPV	Rapamycin + MDPV	Vehicle + MDPV
Day 16–20	Vehicle	Vehicle	Vehicle	Rapamycin
Day 21	MDPV	MDPV	MDPV	MDPV

### Measurement of locomotor activity

Repeated measurement of locomotor activity was used to assess the induction and expression of behavioral sensitization to MDPV, as previously published [36], with minor modifications. Measurements were taken on days 1, 7 and 21 using Opto-Varimex Auto-Track (model 0271-002 M, Columbus Instruments, Columbus, OH, USA) open field chambers (20.3 × 20.3 × 20.3 cm; 4 units) equipped with 16 infrared beams and corresponding photodetectors, spaced by 1.3 cm; located on the X and Y horizontal axes, on two planes, to detect both horizontal and vertical movements.

On each testing day, mice were allowed to habituate for 60 min, after which the proper testing of measurement of locomotor activity was conducted for 120 min. Both habituation and measurement were conducted under a dim red light. On testing days 1 and 7 animals obtained the first injection of rapamycin (10 mg/kg) or vehicle and were immediately placed in the apparatus for habituation. Then they received the second injection of MDPV (1 mg/kg) or saline just before the start of measurement. On day 21 mice were placed in the apparatus for 60 min of habituation, after which obtained a challenge dose of 1 mg/kg of MDPV just before the start of measurement.

Apparatus detects beam breaks every 0.1 s and based on their sequence dedicated software tracks the position (X, Y, Z) of the animal and calculates parameters related to the movement, such as path, speed, distance covered and occurrence of rearing.

### Data analysis

Data were expressed as mean ± standard error of the mean (SEM). All statistical analyses were performed using GraphPad Prism 6 software (GraphPad Software, San Diego, CA, USA). The results were considered statistically significant when *p* < 0.05. Two-way repeated measures ANOVA (treatment × time, or day × time) followed by Sidak’s post hoc test was performed using distance covered (cm) and count of rearing behavior as an index of locomotor activity.

## Results

Statistical analysis revealed that the measures of locomotor activity: distance and rearing on day 1 and day 7 of MDPV (1 mg/kg) administration were significantly affected by the treatment (combination of drugs that each group obtained affected the measures of locomotor activity during the entire observation period), time (measures of locomotor activity of all mice aggregated into 10-min bins changed during the 120-min observation) and treatment × time interaction (pattern of change of measures of locomotor activity in time was different between experimental groups). On day 21 both the distance and rearing activity were significantly affected by the time and treatment × time interaction, but not the treatment itself, as all groups were treated with MDPV on this day. Detailed results of this statistical analysis are presented in Table 2.

**Table 2:** Results of statistical analysis performed between groups on days 1, 7 and 21

	Treatment	Time	Treatment × Time
Day 1 distance	F_3,44_ = 17.30 ***p* < 0.0001**	F_11,484_ = 84.13 ***p* < 0.0001**	F_33,484_ = 5.209 ***p* < 0.0001**
Day 1 rearing	F_3,44_ = 9.806 ***p* < 0.0001**	F_11,484_ = 50.77 ***p* < 0.0001**	F_33,484_ = 4.089 ***p* < 0.0001**
Day 7 distance	F_2,33_ = 15.51 ***p* < 0.0001**	F_11,363_ = 27.20 ***p* < 0.0001**	F_22,363_ = 11.07 ***p* < 0.0001**
Day 7 rearing	F_2,33_ = 10.04 ***p* = 0.0004**	F_11,363_ = 7.945 ***p* < 0.0001**	F_22,363_ = 7.211 ***p* < 0.0001**
Day 21 distance	F_2,33_ = 2.440 *p* = 0.1027	F_11,363_ = 47.37 ***p* < 0.0001**	F_22,363_ = 2.458 ***p* = 0.0003**
Day 21 rearing	F_2,33_ = 2.394 *p* = 0.1070	F_11,363_ = 21.71 ***p* < 0.0001**	F_22,363_ = 1.823 ***p* = 0.0139**

Bold values indicate statistical significance

Apart from differences between groups of mice on each day of measurements, we also analyzed differences in the activity of animals recorded on different days (1, 7, and 21) within each group. For all groups treated with MDPV on days 1–7 both the distance covered and the incidence of rearing activity were significantly affected by the time (during all days of testing measures of locomotor activity aggregated into 10-min bins changed during the 120-min observation) and day × time interaction (pattern of change of measures of locomotor activity of an experimental group was different between the days of testing), but not by the day of the experiment itself (measures of locomotor activity during the entire 120-min observation did not differ between the days of testing). Detailed results of this statistical analysis are presented in Table 3.

**Table 3:** Results of statistical analysis performed within groups on days 1, 7 and 21

	Day	Time	Day × Time
Group 2 distance	F_2,22_ = 2.465 *p* = 0.1081	F_11,121_ = 43.62 ***p* < 0.0001**	F_22,242_ = 3.944 ***p* < 0.0001**
Group 2 rearing	F_2,22_ = 3.418 *p* = 0.051	F_11,121_ = 22.11 ***p* < 0.0001**	F_22,242_ = 2.429 ***p* = 0.0005**
Group 3 distance	F_2,22_ = 2.994 *p* = 0.0708	F_11,121_ = 45.73 ***p* < 0.0001**	F_22,242_ = 2.413 ***p* = 0.0006**
Group 3 rearing	F_2,22_ = 2.505 *p* = 0.1047	F_11,121_ = 26.30 ***p* < 0.0001**	F_22,242_ = 3.742 ***p* < 0.0001**
Group 4 distance	F_2,22_ = 0.2717 *p* = 0.7646	F_11,121_ = 45.43 ***p* < 0.0001**	F_22,242_ = 3.409 ***p* < 0.0001**
Group 4 rearing	F_2,22_ = 1.442 *p* = 0.2580	F_11,121_ = 29.51 ***p* < 0.0001**	F_22,242_ = 3.534 ***p* < 0.0001**

Bold values indicate statistical significance

**Fig. 2:**
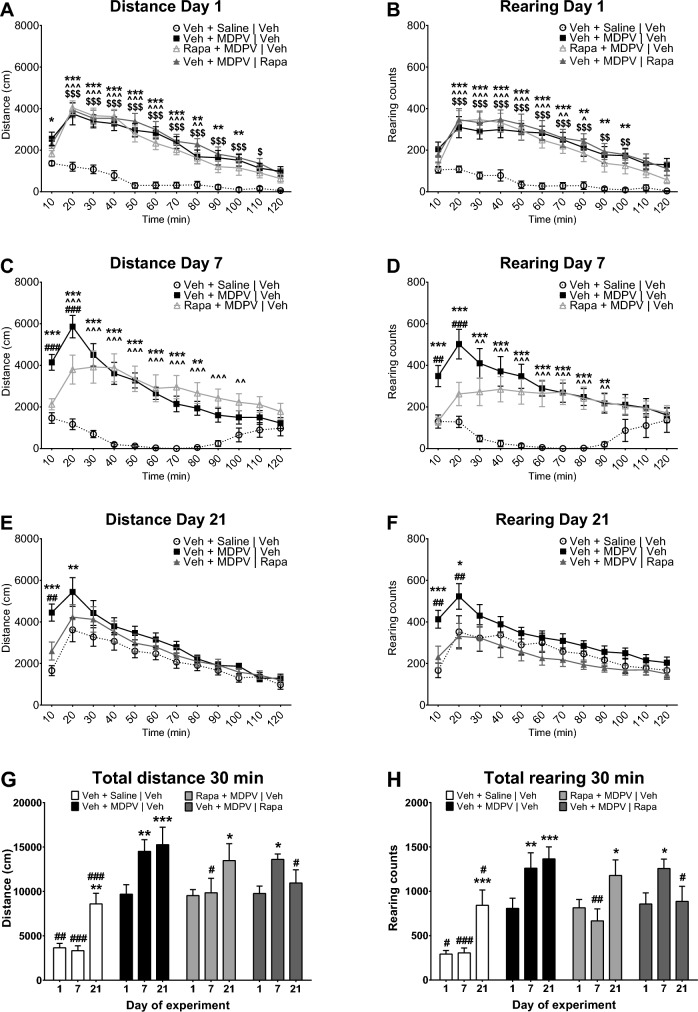
Effects of MDPV (1 mg/kg) and rapamycin (Rapa; 10 mg/kg) on locomotor activity of mice: comparison between groups. Data are presented as mean ± standard error of the mean (SEM), n = 12 per group. **A** Distance covered during 10-min intervals on day 1. **B** Rearing counts in 10-min intervals on day 1. **C** Distance covered during 10-min intervals on day 7. **D** Rearing counts in 10-min intervals on day 7. **E** Distance covered during 10-min intervals on day 21. **F** Rearing counts in 10-min intervals on day 21. ***p < 0.001; **p < 0.01; *p < 0.05 mice treated with vehicle and MDPV on days 1–7 and vehicle on days 16–20 (group 2) vs. mice treated with vehicle and saline on days 1–7 and vehicle on days 16–20 (group 1); ^^^*p* < 0.001; ^^*p* < 0.01; ^*p* < 0.05 mice treated with rapamycin and MDPV on days 1–7 and vehicle on days 16–20 (group 3) vs. mice treated with vehicle and saline on days 1–7 and vehicle on days 16–20 (group 1); $$$*p* < 0.001; $$*p* < 0.01; $*p* < 0.05 mice treated with vehicle and MDPV on days 1–7 and rapamycin on days 16–20 (group 4) vs. mice treated with vehicle and saline on days 1–7 and vehicle on days 16–20 (group 1); ###*p* < 0.001; ##*p* < 0.01 mice treated with vehicle and MDPV on days 1–7 and vehicle on days 16–20 (group 2) vs. mice treated with rapamycin and MDPV on days 1–7 and vehicle on days 16–20 (group 3) (panels **C**, **D**) or mice treated with vehicle and MDPV on days 1–7 and vehicle on days 16–20 (group 2) vs. mice treated with vehicle and MDPV on days 1–7 and rapamycin on days 16–20 (group 4) (panels **E**, **F**). **G** Total distance covered during the first 30 min of measurement. **H** Total rearing counts during the first 30 min of measurement. ***p < 0.001; **p < 0.01; *p < 0.05 vs. day 1 within the same group; ###*p* < 0.001; ##*p* < 0.01, #*p* < 0.05 vs. mice treated with vehicle and MDPV on days 1–7 and vehicle on days 16–20 (group 2) on the same day of experiment

As in every analysis performed interaction played a significant role in the locomotor activities of mice, multiple comparisons were performed for each 10-min time point using Sidak’s post hoc test. On day 1 all mice treated with MDPV (groups 2–4) displayed very similar, strong increases in ambulatory and rearing activities, as illustrated on Fig. 2A, B. Time-curves of the distance and rearing activities of these mice were almost parallel, demonstrating significant increases compared to the saline-treated group during similar periods, which are shown on these figures. Notably, there was no difference at any time-point between any MDPV-treated groups, confirming the homogeneity of psychostimulant effects of MDPV in mice and lack of acute effects of rapamycin.

**Fig. 3:**
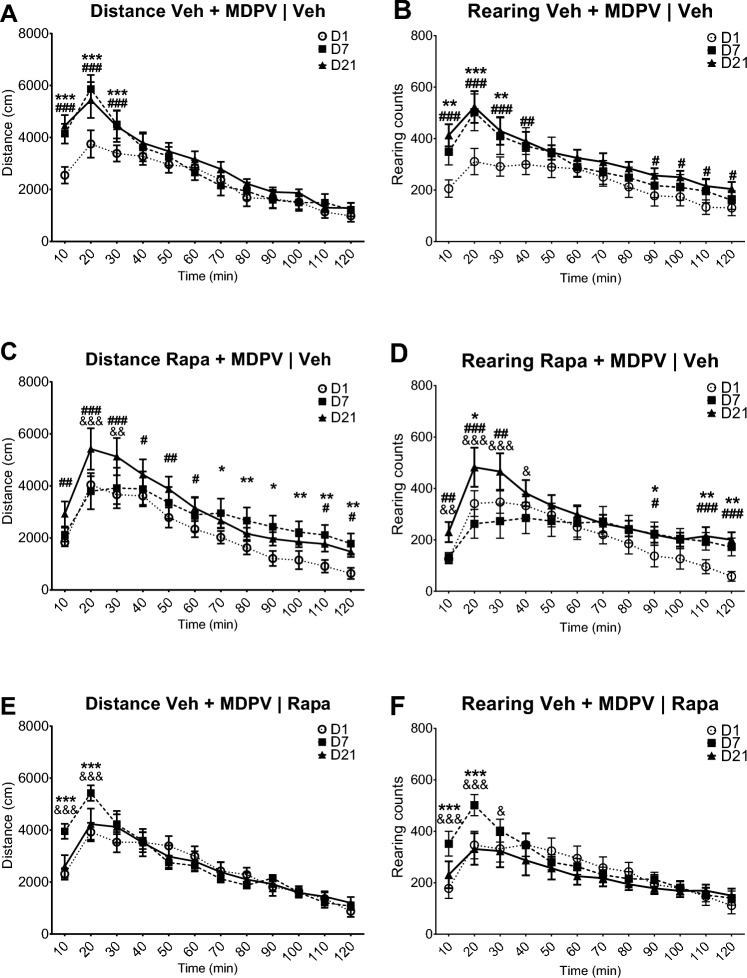
Effects of MDPV (1 mg/kg) and rapamycin (Rapa; 10 mg/kg) on locomotor activity of mice: comparison within groups. Data are presented as mean ± standard error of the mean (SEM), n = 12 per group. **A** Distance covered during 10-min intervals by mice treated with vehicle and MDPV on days 1–7 and vehicle on days 16–20. **B** Rearing counts in 10-min intervals of mice treated with vehicle and MDPV on days 1–7 and vehicle on days 16–20. **C** Distance covered during 10-min intervals by mice treated with rapamycin and MDPV on days 1–7 and vehicle on days 16–20. **D** Rearing counts in 10-min intervals of mice treated with rapamycin and MDPV on days 1–7 and vehicle on days 16–20. **E** Distance covered during 10-min intervals by mice treated with vehicle and MDPV on days 1–7 and rapamycin on days 16–20. **F** Rearing counts in 10-min intervals of mice treated with vehicle and MDPV on days 1–7 and rapamycin on days 16–20. D1—day 1, D7—day 7, D21—day 21. ***p < 0.001; **p < 0.01; *p < 0.05 day 7 vs. day 1; ###*p* < 0.001; ##*p* < 0.01; #*p* < 0.05 day 21 vs. day 1; &&&*p* < 0.001; &&*p* < 0.01; &*p* < 0.05 day 7 vs. day 21

On day 7 mice from the group 2 showed sensitization to the psychostimulant effect of MDPV. During the first 30 min of measurement both their ambulatory and rearing activities were significantly higher compared to day 1 (Fig. 3A, B), an observation indicating the induction of locomotor sensitization. Similarly, during the initial period of measurement locomotor activities of mice from the group 2 were significantly higher as compared to mice from the group 3 treated with rapamycin on days 1–7 (Fig. 2C, D). Likewise, both the horizontal and vertical activities of mice from the group 3 were not increased compared with day 1 during the initial phase of recording (Fig. 3C, D), an observation indicating lack of induction of sensitization.

On day 21 all mice were challenged with 1 mg/kg of MDPV. The group 2 displayed significantly higher horizontal and vertical locomotor activities in the early phase of measurement as compared to day 1 (Fig. 3A, B), indicating expression of the previously induced sensitization. In the initial period of observation, both the horizontal and vertical activities of mice from the group 2 were significantly higher compared to the group 1, which was treated with MDPV on that day for the first time, and compared to the group 4 treated with rapamycin on days 16–20 (Fig. 2E, F). The ambulatory and rearing activities of mice from the group 4 were not significantly different on day 21 compared to day 1 at any time-point of measurement, but were lower during the initial period as compared to day 7 (Fig. 3E, F), indicating abolishment of the previously established sensitization.

As in the group 2 the increased behavioral response to MDPV on day 7 and day 21 was observed mostly during the first 30 min, total horizontal and vertical locomotor activities of mice during the initial 30 min of the observation were further analyzed. Two way-ANOVA revealed that the treatment (F_3,44_ = 13.72; *p* < 0.0001), day (F_2,88_ = 14.78; *p* < 0.0001) and interaction (F_6,88_ = 3.593; *p* = 0.0031) significantly affected the distance covered by mice. Similarly, the treatment (F_3,44_ = 7.372; *p* = 0.0004), day (F_2,88_ = 14.34; *p* < 0.0001) and interaction (F_6,88_ = 5.583; *p* < 0.0001) also significantly affected the total rearing activity of mice (Fig. 2G, H).

The group 2, which received vehicle during both periods and MDPV (1 mg/kg) before each measurement, successfully developed sensitization. Both the total distance covered and the count of rearing activity were higher on day 7 and day 21 compared to day 1 (Fig. 2G, H). Acute treatment with rapamycin (10 mg/kg) did not affect the response of mice to MDPV (1 mg/kg), as during day 1 no significant differences were observed in the distance covered or rearing activity between the group 2, which obtained MDPV after pretreatment with vehicle, and the group 3, which received MDPV after pretreatment with rapamycin (Fig. 2G, H).

Pretreatment with rapamycin during the induction phase (group 3) abolished the induction of locomotor sensitization to effects of MDPV. On day 7 mice from the group 3 did not display increase in the distance covered or the occurrence of rearing behavior compared to day 1. Moreover, both parameters were significantly lower compared to the group 2 on day 7 (Fig. 2G, H). Interestingly, when rapamycin was not administered during the period of withdrawal from MDPV, both the distance covered and the rearing activity in the group 3 were significantly greater on day 21 compared to day 1, an observation indicating expression of locomotor sensitization after the abstinence (Fig. 2G, H).

During days 1–7 the group 4 did not differ from the group 2 in terms of the treatment. Consequently, data from day 7 are similar between these groups, as in both cases there is a significant increase of the distance covered and the occurrence of rearing compared to day 1, an observation confirming the induction of sensitization (Fig. 2G, H). Administration of rapamycin (10 mg/kg) during days 16–20 resulted in abolishment of expression of sensitization to MDPV, as on day 21, after a challenge dose of this drug, mice from the group 4 covered a shorter distance and performed a smaller number of rearing activity compared to the group 2; both parameters did not differ from the results obtained on day 1 in the group 4 (Fig. 2G, H).

## Discussion

The aim of the current study was to examine effects of rapamycin, the selective and highly potent inhibitor of mTOR [37], on the early stage of addiction to the psychostimulant synthetic cathinone MDPV, using behavioral model of this process, i.e., the locomotor sensitization paradigm. Repeated treatment of rodents with psychostimulants causes stimulation of locomotor activities with a potency increasing during the treatment period and after withdrawal. This phenomenon is called sensitization, and is considered a behavioral marker of an early stage of addiction [11]. Generally, the process of sensitization involves three basic consecutive stages: an initial induction phase followed by a drug withdrawal, and then a re-exposure to the drug to allow the expression of sensitization. Several studies have demonstrated that the process of behavioral sensitization is mediated by neuroadaptive changes in the mesolimbic dopaminergic system, and involves relative changes in the function of dopamine receptors (D1-DR: D2-DR ratio) – resulting from altered expression and sensitivity of primarily D2-DR, and in expression and activity of DAT in axons of dopaminergic neurons projecting from the VTA to the NAc, a part of ventral striatum and key anatomical structure of the reward system [29, 38–41].

In our experimental setting mice treated with MDPV developed sensitization, as their peak response to the drug significantly increased after 7 days of consecutive treatment with the drug and then after a withdrawal period lasting for 13 days. Rapamycin administered during the induction phase attenuated the increase of the peak response to MDPV on day 7, however the inhibitory effect of rapamycin did not persist until the MDPV challenge on day 21. Additionally, treatment with rapamycin during the final 5 days of abstinence from MDPV was sufficient to abolish the expression of sensitization. Our results are in line with those reported by other researchers. Wu and colleagues [26] observed that rapamycin administered either during daily treatment of rats with cocaine or during the withdrawal period was able to completely abolish the sensitization on the challenge day. Furthermore, the inhibitory effect of rapamycin on sensitization to cocaine in C57BL/6J mice was seen on the challenge day in animals that were treated with rapamycin during the withdrawal period [30]. Anti-addictive properties of rapamycin have been also observed in studies on rodents using other behavioral models. For instance, rapamycin attenuated the expression of cocaine-induced place preference in mice [25], reduced cocaine-seeking behavior and prevented relapse in experiments using self-administration paradigm in rats [33].

We did not observe acute effects of rapamycin on the MDPV-induced locomotor activity in mice on day 1 (Fig. 2A, B, G, H). This is in line with other published studies, in which mice treated with saline and rapamycin did not differ in their locomotor activity from the control group through the whole experimental schedule [25, 26, 30]. Thus, it can be concluded that rapamycin itself does not affect locomotor activity of rodents neither after acute nor after repeated administration. Based on those previously published studies we decided not to include groups treated with saline and rapamycin in the current study, in order to reduce the number of animals used for procedures.

mTOR modulates neuroplastic changes by forming mTORC1 complex which regulates expression and activity of numerous targets, including among others 4E-BP1, S6K which phosphorylates ribosomal S6 protein (S6), and synapse differentiation-induced gene 1 (SynDIG1). Recent studies indicate that psychostimulants, such as cocaine, activate mTORC1 and its downstream signaling pathways in addiction-related brain structures, notably NAc, VTA, and PFC [26, 28–30, 42]. Through this mechanism mTORC1 regulates translation of various proteins involved in synaptic plasticity (comprising of axon growth and navigation, arborization of dendrites, changes in neuronal morphology, formation of new synapses, changes of expression of receptors and channels), memory formation and adaptive changes related to addiction. Those proteins include, among others, Ca^2+^/Calmodulin-dependent kinase II alpha (CamKIIα), AMPA receptor subunits (GluRs), post-synaptic density protein 95 (PSD-95) and glutamate receptor interaction protein [26, 28, 30]. Taken together, mTORC1 may be viewed as a regulator of glutamatergic and dopaminergic systems, both of which play a role in addiction-related behavioral changes [26, 43–45]. Particularly, changes in surface expression of AMPA receptor subunits, including GluR1, GluR2, and GluR3 in NAc [25, 30], due to changes in their intracellular translocation were observed after withdrawal from cocaine and are considered responsible for sensitization and drug seeking [25, 30]. Those changes include increased surface/intracellular ratios for GluR1 and GluR2/3 subunits [46] and decreased presence of AMPA receptors containing GluR2 subunit [30, 47]. Interestingly, rapamycin was found to inhibit cocaine-induced phosphorylation of S6 in brain structures of rat [26], leading to suppression of locomotor sensitization to cocaine without affecting acute locomotor effect of the drug [25, 26]. Rapamycin also decreased surface expression of AMPA receptor GluR2/3 subunits in stimulated cultured neurons [48]. Thus, it is reasonable to assume that, by inhibition of mTORC1 activation, rapamycin prevents sensitization to MDPV by inhibition of above-mentioned downstream pathways and resulting translational changes. Unfortunately, we did not investigate the aforementioned targets to support such hypothesis.

The current study has some limitations that should be accounted for. Using the current protocol, we were able to detect locomotor sensitization in the group 2, defined as an increase in peak locomotor activity on days 7 and 21 compared to day 1, during a relatively short period, i.e., during the initial 30 min of the measurement. However, a similar response was observed in rats treated with 0.5 mg/kg MDPV—a significant increase in the number of beam breaks between day 7 and day 1 was observed only in the first 25 min after the drug administration [13]. The dose 1 mg/kg used in the current study was selected based on our experience with measurement of locomotor activity in C57BL/6 J mice treated with various SC [36, 49]. In our previous experiments [36] mice treated with MDPV at 1 mg/kg covered at average approx. 23000 cm during 120 min. Using other compounds such as pyrovalerone or α-PVP, although at higher doses, total average distances of approx. 46500 cm and 48500 cm could be observed during 120 min of observation, respectively [36, 49]. Therefore, it appeared that dose of 1 mg/kg leaves a wide margin for a significant increase. On the other hand, MDPV at 1 mg/kg dose caused a significant increase of horizontal locomotor activity compared to the control group within the period of 10–120 min post injection, while the higher dose of 3 mg/kg caused an increase during 0–120 min post injection [36]. However, the additional analysis of those data indicates that the horizontal activity of mice treated with 3 mg/kg of MDPV is significantly higher compared to MDPV at 1 mg/kg only during 0–20 min post injection period [36]. Thus, it appears that increasing potency of the effect of MDPV on locomotor activity of rodents, either due to a higher dose or due to sensitization could be observed mainly in the initial period of the measurement. Accordingly, mice treated with rapamycin during the induction phase (group 3) demonstrated markedly weaker peak response to MDPV on day 7, compared to animals treated with MDPV and vehicle (group 2). Similarly, during the MDPV challenge on day 21, mice treated with rapamycin during the withdrawal phase (group 4) demonstrated the weaker peak response to MDPV when compared to animals treated with MDPV and vehicle (group 2). In both cases differences in locomotor activities of groups 3 and 4 compared to group 2 disappeared in the later part of experiment. Such outcomes may result from the relatively high dose of MDPV (1 mg/kg) used in the present study. At 1 mg/kg, MDPV evoked very strong stimulation of locomotor activities of mice, starting from the first day, leaving relatively little space for further increase of the response. It appears likely that persistence of such high stimulation of locomotor activity as was seen during the first 30 min of measurement in the group 2 on day 7 and day 21 is beyond physical capabilities of mice, due, for example, to fatigue. Another explanation of this observation was proposed by Berquist and colleagues [13], who suggested that on consecutive days of measurement the novelty of the open-field chambers is decreased, thus animals have lesser desire to explore after initial strong stimulation. While other factors contributing to observed pattern of response to MDPV, such as pharmacodynamic or pharmacokinetic properties of the drug, cannot be ruled out, we do not have any evidence to confirm them. Use of a lower dose of MDPV could possibly lead to a better separation of response-curves between days and treatments, resulting in a higher sensitivity to detect both sensitization (i.e., increased response during longer period) and inhibitory effect of rapamycin (i.e., significant differences between groups during longer period).

Other limitation of the current study is that locomotor sensitization, serving as a model of early phase of addiction, was the only behavioral method utilized and that no molecular assays were performed to reveal the mechanism by which rapamycin prevents sensitization to MDPV. Future studies utilizing behavioral tests such as conditioned place preference, drug self-administration and discrimination would provide more data on the effects of inhibition of mTOR by rapamycin on other markers of addiction to MDPV, including rewarding effects, drug-seeking and its relapse. Additionally, changes of expression and activity of targets relevant to addiction, such as components of dopaminergic system, AMPA receptor subunits and markers of synaptic plasticity in PFC and NAc are warranted to better understand the complex effects of mTOR inhibition in the nervous system of animals exposed to MDPV. This is of particular importance, as it was previously documented using other psychoactive substances, that rapamycin abolishes those markers of addiction in laboratory animals via interaction with above-mentioned molecular targets [25–34].

Findings of the current study demonstrate that inhibition of mTOR by rapamycin blocks the induction and the expression of behavioral sensitization to psychostimulant β-cathinone derivative MDPV. Although further research is needed to fully unravel the molecular mechanism of abolishment of sensitization by rapamycin, inhibition of mTOR emerges as an attractive possible target for future therapies of addiction to MDPV and similar substances. Of note, rapamycin counteracted expression of sensitization when it was administered during withdrawal from MDPV. This is of particular importance as it suggests that mTOR inhibitors may be a valuable tool used to facilitate the maintenance of drug abstinence in the therapy of addiction, while currently there is no approved effective pharmacotherapy for addiction to psychostimulants.

## Conclusions

Repeated treatment with MDPV (1 mg/kg) caused behavioral sensitization in C57BL/6JRj mice. Pre-treatment with rapamycin (10 mg/kg), inhibitor of mTOR, successfully prevented induction of sensitization to MDPV, while treatment with rapamycin during the final 5 days of withdrawal abolished expression of sensitization. This suggests that blockade of mTOR interfered with neuroadaptive changes evoked by MDPV and warrants further research on the possible application of mTOR inhibitors in the treatment of addiction.

## Data Availability

The raw data supporting the conclusions of this article will be made available by the authors on request.

## Abbreviations

4E-BP1: Eukaryotic initiation factor 4E-binding protein 1
AMPA: α-Amino-3-hydroxy-5-methyl-4-isoxazolepropionic acid
ANOVA: Analysis of variance
BDNF: Brain-derived neurotrophic factor
CamKIIα: Ca^2+^/Calmodulin-dependent kinase II alpha
D1-DR: Dopamine receptor D1
D2-DR: Dopamine receptor D2
DAT: Dopamine transporter
eIF4B: Eukaryotic initiation factor 4B
GluR1: AMPA receptor subunit 1
GluR2: AMPA receptor subunit 2
GluR3: AMPA receptor subunit 3
MDPV: 3,4-Methylenedioxypyrovalerone
mTOR: Mammalian target of rapamycin
NAc: Nucleus accumbens
NPS: New psychoactive substances
NET: Norepinephrine transporter
PFC: Prefrontal cortex
PSD-95: Post-synaptic density protein 95
Rapa: Rapamycin
SC: Synthetic cathinones
SERT: Serotonin transporter
SynDIG1: Synapse differentiation-induced gene 1
S6: Ribosomal S6 proteins
S6K: P70 ribosomal S6 protein kinase
VTA: Ventral tegmental area
